# Supplementary material for: Enhanced Storage Performance of PANI and PANI/Graphene Composites Synthesized in Protic Ionic Liquids
Source: Materials (Basel). 2021 Jul 30;14(15):4275. doi: 10.3390/ma14154275 (PMC8347535; doi:10.3390/ma14154275)
Supplement: Supplementary file 1 [file materials-14-04275-s001.zip › materials-1261154-supplementary.pdf]

materials

Supplementary Materials

Enhanced Storage Performance of PANI and PANI/Graphene Composites Synthesized in Protic Ionic Liquids

Fatima Al-Zohbi [1], Fouad Ghamouss [1,*], Bruno Schmaltz [1], Mohamed Abarbri [1], Mustapha Zaghrioui [2] and François Tran-Van [1,*]

[1] Laboratoire de Physico-Chimie des Matériaux et des Electrolytes Pour l'Energie (EA 6299), University of Tours, Parc de Grandmont, 37200 Tours, France; alzohbi-fatima@hotmail.com (F.A.-Z.); bruno.schmaltz@univ-tours.fr (B.S.); mohamed.abarbri@univ-tours.fr (M.A.)
[2] CNRS, CEA, INSA CVL, GREMAN UMR 7347, University of Tours, IUT de Blois 15 rue de la Chocolaterie, CS 32903, 41029 Blois, France; mustapha.zaghrioui@univ-tours.fr
* Correspondence: fouad.ghamouss@univ-tours.fr (F.G.); francois.tran@univ-tours (F.T.-V.); Tel.: +33-02-47366923 (F.G.); Tel.: +33-02-47366923 (F.T.-V.)

Citation: Al-Zohbi, F.; Ghamouss, F.; Schmaltz, B.; Abarbri, M.; Zaghrioui, M.; Tran-Van, F. Enhanced Storage Performance of PANI and PANI/Graphene Composites Synthesized in Protic Ionic Liquids. *Materials* **2021**, *14*, 4275. https://doi.org/10.3390/ma14154275

Academic Editor: Alessandro Dell'Era

Received: 31 May 2021
Accepted: 3 July 2021
Published: 30 July 2021

Publisher's Note: MDPI stays neutral with regard to jurisdictional claims in published maps and institutional affiliations.

Copyright: © 2021 by the authors. Licensee MDPI, Basel, Switzerland. This article is an open access article distributed under the terms and conditions of the Creative Commons Attribution (CC BY) license (http://creativecommons.org/licenses/by/4.0/).

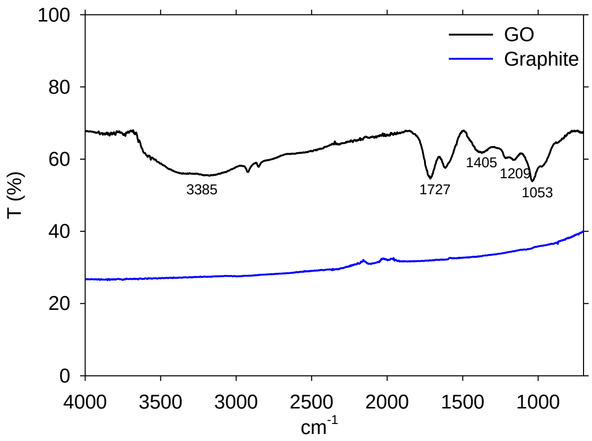

Figure S1. ATR- FTR spectra of GO and graphite.

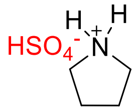

Figure S2. Chemical structure of [Pyrr][HSO_4].

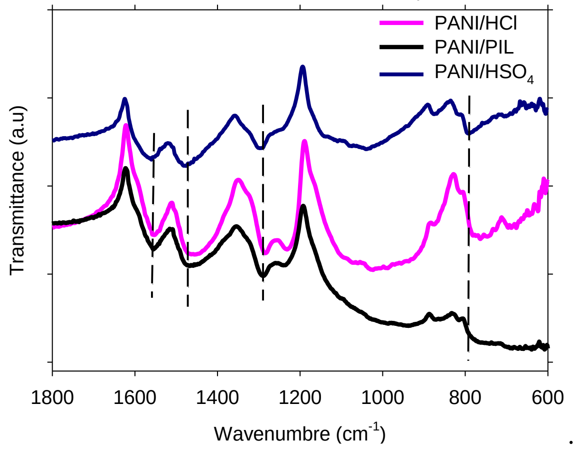

Figure S3. ATR- FTR spectra of PANI/HCl, PANI/PIL and PANI/HSO_4.

Table S1. Main ATR-FTIR bands of synthesized PANI.

Wavenumber (cm^{-1})	Band Characteristics
776	Para-disubstituted aromatic ring [1,2]
1028	C-H in plane bending vibration [3]
1238	C-$N^{+\cdot}$ stretching [4]
1295	Aromatic C-N stretching indicating secondary aromatic amine group [3]
1466	C-N strecting of benzenoid rings [3]
1558	C-N stretching of quinoid rings [3]

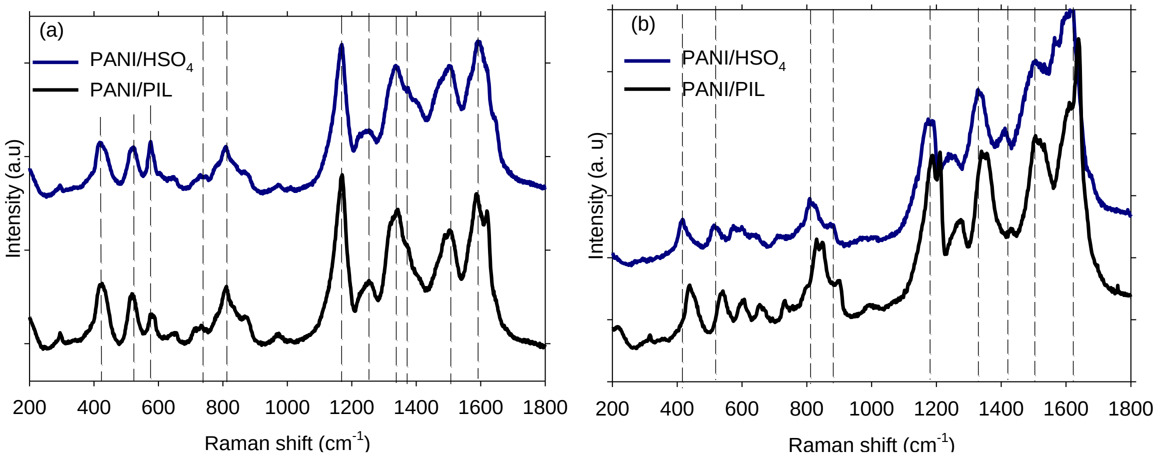

Figure S4. Raman spectra of Pani/PIL and Pani/HSO_4 using **(a)** 633 and **(b)** 514 nm excitations.

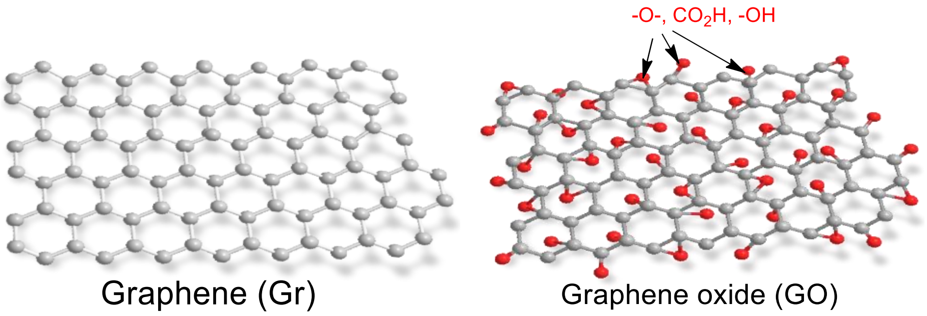

Figure S5. Graphene (Gr) *vs.* graphene oxide (GO).

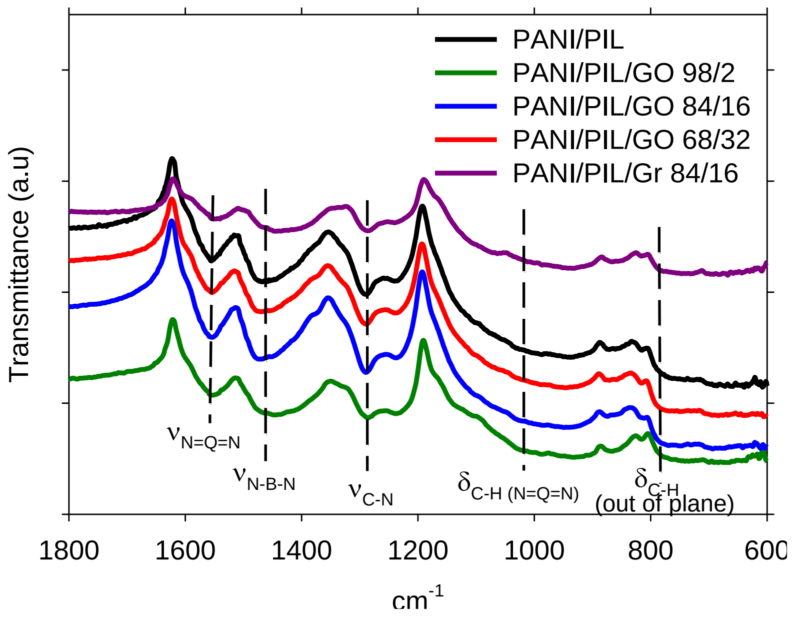

Figure S6. AT-IR spectra of the PANI/HCl, PANI/PIL and PANI/PIL/GO nanocomposites (B : benzoid units, Q : quinonid units, ◎: stretching vibration and◎◎: bending vibration).

Table S2. Specific capacitance, energy and power densities at 10 A.g^{-1} of PANI/HCl, PANI/PIL and PANI/PIL/GO 84/16.

Electrode Material	ATR-FIR (F.g^{-1}) @ 10 A.g^{-1}	Energy Density Wh.Kg^{-1} @ 10 A.g^{-1}	Power Density W.Kg^{-1} @ 10 A.g^{-1}
PANI/HCl	65.8	1.46	2930
PANI/PIL	173.4	3.85	2942
PANI/PIL/GO 84/16	223.0	4.95	3687

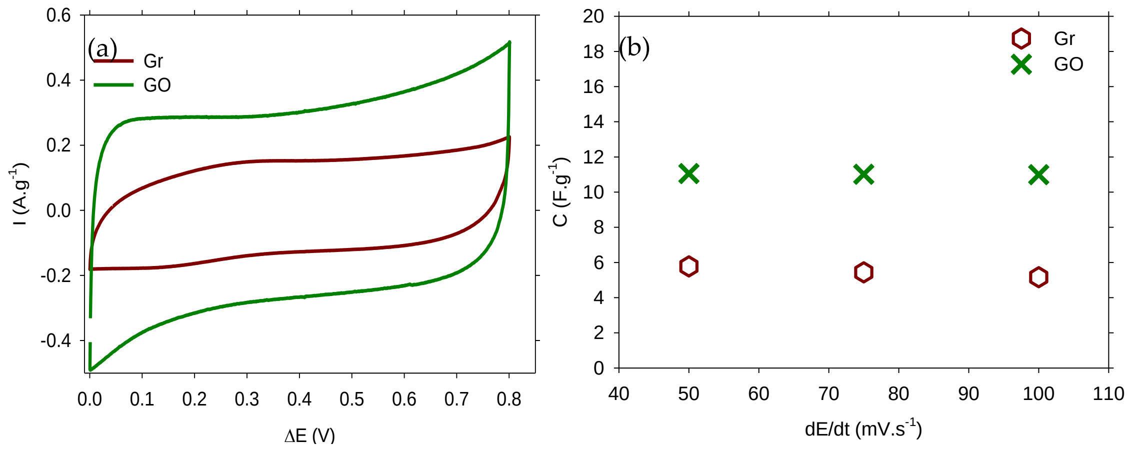

Figure S7. (**a**) CV curves at 100 mV.s^{-1} and (**b**) specific capacitance as function of scan rate of Gr and GO in H_2SO_4 1 mol.L^{-1}. CVs have been recorded using two electrodes configurations (symmetric capacitors). Specific capacitances are relative to the total weight of the electrodes in the symmetric capacitors.

References

1. Kulkarni MV, Viswanath AK, Aiyer RC, Khanna PK. Synthesis, characterization, and morphology of p-toluene sulfonic acid-doped polyaniline: A material for humidity sensing application. J *Polym Sci, Part B: Polym Phys.* **2005**; 43:2161-9.
1. Jayamurugan P, Mariappan R, Ponnuswamy V, Manikandan H, Asokan S, Saravanan S. High-PL efficiency of polyaniline using various dopants. *Optik* **2011**; 122:2083-5.
2. Stejskal J, Sapurina I, Trchová¡ M, Prokeš J, Křivka I, Tobolková E. Solid-State Protonation and Electrical Conductivity of Polyaniline. *Macromolecules* **1998**; 31:2218-22.
3. Quillard S, Louam, G., Buisson JP, Boyer, M., Lapkowski M, Pron, A., Lefrant S. Vibrational spectroscopic studies of the isotope effects in polyaniline. *Synth Met*. **1997**; 84:805-6.
